# Association of *IRS1* gene Pro512Ala polymorphism with nonalcoholic fatty liver disease

**DOI:** 10.20945/2359-4292-2023-0216

**Published:** 2024-08-26

**Authors:** Asadollah Asadi, Mitra Rostami, Radmehr Shafiee, Abbas Ardalani, Atefeh Dehghanitafti, Zakieh Golshadi, Kiarash Kohansal, Fatemeh Ghasemi, Maryam Najafi, Touraj Mahmoudi, Gholamreza Rezamand, Reza Dabiri, Hossein Nobakht, Hamid Farahani, Seidamir Pasha Tabaeian

**Affiliations:** 1 University of Mohaghegh Ardabili Faculty of Science Department of Biology Ardabil Iran Department of Biology, Faculty of Science, University of Mohaghegh Ardabili, Ardabil, Iran; 2 Shahid Beheshti University of Medical Sciences Research Institute for Gastroenterology and Liver Diseases Gastroenterology and Liver Diseases Research Center Tehran Iran Gastroenterology and Liver Diseases Research Center, Research Institute for Gastroenterology and Liver Diseases, Shahid Beheshti University of Medical Sciences, Tehran, Iran; 3 Tehran University Faculty of Veterinary Medicine Department of Clinical Pathology Iran Department of Clinical Pathology, Faculty of Veterinary Medicine, Tehran University, Iran; 4 Iran University of Medical Sciences Physiology Research Center Tehran Iran Physiology Research Center, Iran University of Medical Sciences, Tehran, Iran; 5 Iran University of Medical Sciences School of Medicine Department of Internal Medicine Tehran Iran Department of Internal Medicine, School of Medicine, Iran University of Medical Sciences, Tehran, Iran; 6 Iran University of Medical Sciences Colorectal Research Center Tehran Iran Colorectal Research Center, Iran University of Medical Sciences, Tehran, Iran; 7 Semnan University of Medical Sciences Internal Medicine Department Semnan Iran Internal Medicine Department, Semnan University of Medical Sciences, Semnan, Iran; 8 Qom University of Medical Sciences School of Medicine Department of Physiology and Pharmacology Qom Iran Department of Physiology and Pharmacology, School of Medicine, Qom University of Medical Sciences, Qom, Iran

**Keywords:** Insulin, IRS1, NAFLD, polymorphism, variant

## Abstract

**Objective:**

This study was designed to investigate the possible effect of the insulin receptor substrate 1 *(IRS1)* gene rs1801276 polymorphism on the risk of nonalcoholic fatty liver disease (NAFLD).

**Subjects and methods:**

The rs1801276 polymorphism was investigated in 127 controls and 123 biopsy-proven NAFLD patients using PCR-RFLP.

**Results:**

No deviation from Hardy-Weinberg equilibrium was discovered for the rs1801276 variant of *IRS1* in either NAFLD patients or controls (*P*>0.05). The distribution of different rs1801276 genotypes and alleles showed significant variations between controls and NAFLD patients. In comparison to rs1801276 ‘CC’ genotype, the "GG+GC" genotype occurred less frequently in NAFLD patients than in controls, which also persisted after adjustment for confounding factors (*P* = 0.041, OR = 0.60, 95% CI = 0.45-0.93). In comparison with the *IRS1* rs1801276 "C" allele, the "G" allele was significantly less prevalent in NAFLD patients than in controls (*P* = 0.045, OR = 0.69, 95% CI = 0.58-0.91).

**Conclusions:**

For the first time, we reported a significant association between the *IRS1* rs1801276 polymorphism and biopsy-proven NAFLD. More studies are required to further elucidate the contribution of the *IRS1* gene to NAFLD susceptibility.

## INTRODUCTION

Nonalcoholic fatty liver disease (NAFLD), a chronic disease and a global epidemic, is characterized by hepatocellular accumulation of triglycerides in the liver without inordinate alcohol intake. NAFLD constitutes a range of liver lesions from simple steatosis or nonalcoholic fatty liver to inflammation, steatohepatitis, fibrosis, and cirrhosis. In spite of the fact that NAFLD affects approximately 25% of the adult population in the world, the exact underlying molecular mechanisms of its pathogenesis are poorly established (1). However, prior literature shows that insulin resistance (IR) (2,3) and obesity (4) are usually among the hallmarks of NAFLD. NAFLD has a close association with type 2 diabetes (T2D) (4), high serum insulin level (5), abnormal glucose tolerance (6), visceral fat accumulation (2), dyslipidemia (4), and hypertension (4) too. Alterations in lipid metabolism by peripheral IR can cause obesity, which in turn induces hepatic IR and the development of NAFLD (3). Consistent with these findings, the levels of liver enzymes are higher in NAFLD patients with IR versus those without IR (7), and subjects with nonalcoholic steatohepatitis (NASH) have higher IR intensity than those with nonalcoholic fatty liver (8).

Insulin receptor substrate 1 (IRS1) which is widely expressed in human tissues is an endogenous substrate for insulin receptor (INSR) and a docking protein between the INSR and its downstream kinases. IRS1 also transmits signals from insulin like growth factor 1 (IGF1) receptor (IGF1R) to effector proteins and this way it can play a role in regulation of the metabolic actions of IGF1. Phosphorylated by INSR tyrosine kinase, IRS1 plays a key role in transduction of insulin signaling, which in turn controls glucose and lipid metabolism. In fact, IRS1 seriously participates in the regulation of insulin secretion by pancreatic β-cells, insulin action, peripheral insulin sensitivity, and modulating tissue response to insulin (9,10). The surplus of free fatty acids causes hepatic IR through down regulating IRS1 and activation of nuclear factor kappa B (NF-κB) signaling pathways (10). Interestingly, significant associations have been discovered between *IRS1* gene SNPs and serum levels of insulin (11) and IR (11,12). Finally, some variants in other insulin pathway related genes have been reported to be associated with NAFLD (5,13,14). In attempting to understand the possible role of IRS1 in NAFLD, we conducted a case-control study to explore the association between the rs1801276 or Pro512Ala polymorphism of the *IRS1* gene and NAFLD. Here we selected the missense variant of Pro512Ala on the basis of relatively high degree of heterozygosity, high usage frequency in earlier research, and location in the gene (exon 1).

## SUBJECTS AND METHODS

### Study participants

In the present case-control study we recruited 250 Iranian and genetically unrelated subjects including 127 controls (age range, 30-72 years) and 123 biopsy-proven NAFLD patients (age range, 33-76 years). The subjects were recruited consecutively, with the intention of being representative of their respective populations (*i.e.*, no selection bias). The present study complied with ethical standards specified by the Declaration of Helsinki and its amendments. The study was approved by the Ethics Committee of the Institute (Shahid Beheshti University of Medical Sciences) too. The clinical data of the participants were collected by self-administered questionnaires after being informed about the study's aim and obtaining their consent. The diagnostic criteria for NAFLD were as follows: (a) identification of hepatic steatosis on ultrasonography (b) elevated circulating liver enzymes (c) the absence of secondary causes of hepatic fat accumulation such as heavy alcohol consumption (more than 70 g for women or 140 g for men per week), viral hepatitis B and C, Wilson's disease, autoimmune liver disease, or drug-induced liver injury (d) liver biopsy consistent with NAFLD by an experienced pathologist whose analyses of the biopsy samples accorded with the Brunt's criteria. In this study, percutaneous liver biopsy as the most common method of liver biopsy was used. Biopsies were taken from the right liver lobe with Tru-Cut biopsy needles (16 gauge). The length and diameter of the liver samples were 1-2 cm and 1.0-1.5 mm, respectively. According to the scoring system, the grades of steatosis and necroinflammation were from 0 to 3, and the stages of fibrosis were from 0 to 4 (15). We recruited the controls from people who had the same geographical origin of the NAFLD patients, and they were students of Shahid Beheshti University of Medical Sciences or the staff of Research Institute for Gastroenterology and Liver Diseases. The control group was composed of subjects without abnormalities on abdominal ultrasound imaging and they all had normal serum levels of liver enzymes. None of them had viral hepatitis infection or were alcoholic or took regular medications too. The formula for calculation of BMI was weight divided by height squared.

### Genotyping

All the laboratory assistants who performed the experiments were blind to the data of the subjects. Five millimeter of blood samples was taken into separate tubes containing EDTA as anti-coagulant and stored at 4 °C until DNA isolation. Genomic DNA extraction from blood samples was carried out by using phenol chloroform extraction and ethanol precipitation protocol. DNA samples stored at -20 °C until use. The *IRS1* genotyping was conducted using polymerase chain reaction-restriction fragment length polymorphism (PCR-RFLP) analysis. Briefly, genomic DNA was amplified using the primers: 5’-GTCTGTCGTCCAGTAGCAC-3’ and 5’-ATCTCTGTGTACTCCTCAATGG-3’ to detect the genotypes of the *IRS1* rs1801276 variant. PCR was performed with the following program: (a) pre-denaturation at 95 °C for 10 min (b) 35 cycles of denaturing at 95 °C for 40s, annealing at 61 °C for 40s, and extension at 72 ºC for 45s (c) final extension at 72 ºC for 8min. The amplified products were then analyzed by RFLP. After overnight digestion of the PCR product (491 bp) at 37 ºC in a water bath with the restriction enzyme of DraIII (Fermentas, Leon-Rot, Germany), the RFLP products (491 bp, 362 bp, and 129 bp) were electrophoresed on 3% agarose gel and stained with ethidium bromide for visualization using a UV transilluminator. The "G" allele of the *IRS1* rs1801276 SNP had bands of 362 bp and 129 bp, whereas its "C" allele had a band of 491 bp, thus an individual with band(s) at 362 and 129 bp, at 491 bp only, and at 491, 362 and 129 bp was defined as GG homozygotic genotype, CC homozygotic genotype, and GC heterozygotic genotype, respectively. For evaluating the reproducibility of the genotyping results, 15% of all the samples was randomly selected and genotyped again; the reproducibility was 100%.

### Statistical analyses

To conduct statistical analyses, we used the SPSS software package for Windows, version 25.0. We employed t-test to compare continuous data which were presented as mean (standard deviation) and used chi-square (χ^2^) test to compare categorical clinical variables which were expressed as number (percent). The Hardy-Weinberg equilibrium (HWE) for the *IRS1* gene Pro512Ala polymorphism was also verified using χ^2^ test in the patient and control groups separately. This test was used to assess the possible difference in allele frequencies between the control and case groups too. Logistic regression analysis was applied for appraising the association between the genotype frequencies and NAFLD risk as well as adjusting confounding factors. To appraise the strength of the associations, we computed the odds ratios (ORs) with their corresponding 95% confidence intervals (95% CIs). Differences in biochemical parameters among the different *IRS1* genotypes were tested by analysis of variance (ANOVA) and analysis of covariance (ANCOVA) when appropriate. A *P*-value less than 0.05 was taken to be statistically significant.

## RESULTS

### Clinicopathological and biochemical analysis

The general characteristics of the participants are depicted in Table 1. In comparison to the controls, the patients with NAFLD were more likely to be male (*P* < 0.001) and smoker (*P* = 0.027) and had higher age (*P* < 0.001), BMI (*P* < 0.001), systolic blood pressure (*P* < 0.001), diastolic blood pressure (*P* < 0.001), aspartate aminotransferase (*P* < 0.001), alanine aminotransferase (*P* < 0.001), and gamma glutamyl transferase (*P* < 0.001).

**Table 1:** Demographic, anthropometric, and laboratory features of nonalcoholic fatty liver disease (NAFLD) patients and controls^a^

Characteristics		Controls (n = 127)	Patients (n = 123)	P-value
Age (years)		30.1 (6.8)	37.3 (8.7)	<0.001
Body mass index (kg/m^2^)		24.2 (3.6)	28.4 (5.1)	<0.001
Sex				
	Male	68 (53.5)	84 (68.3)	
	Female	59 (46.5)	39 (31.7)	<0.001
Smoking history				
	No	110 (86.6)	94 (76.4)	
	Former	6 (4.7)	13 (10.6)	
	Current	11 (8.7)	16 (13.0)	0.027
Systolic blood pressure (mmHg)		115.2 (14.0)	125.2 (16.3)	<0.001
Diastolic blood pressure (mmHg)		70.2 (8.7)	73.7 (8.8)	<0.001
Aspartate aminotransferase (IU/L)		21.4 (8.0)	40.2 (18.4)	<0.001
Alanine aminotransferase (IU/L)		20.8 (9.9)	69.2 (39.5)	<0.001
Gamma glutamyl transferase (IU/L)		20.1 (9.2)	56.5 (30.3)	<0.001
Steatosis				
	Grade 0		-	
	Grade 1		27 (21.9)	
	Grade 2		68 (55.3)	
	Grade 3		28 (22.8)	
Necroinflammation				
	Grade 0		32 (26.0)	
	Grade 1		50 (40.7)	
	Grade 2		39 (31.7)	
	Grade 3		2 (1.6)	
Fibrosis				
	Stage 0		65 (52.8)	
	Stage 1		51 (41.5)	
	Stage 2		6 (4.9)	
	Stage 3		1 (0.8)	
	Stage 4		-	

a Variables presented as mean (standard deviation) or number (percent); T-test was used to compare continuous data which are presented as mean (standard deviation) and chi-square test was employed to compare categorical variables which are expressed as number (percent).

### *IRS1* gene polymorphism analysis

Table 2 represents genotype and allele frequencies of the *IRS1* gene rs1801276 polymorphism among the control and patient populations. No deviation from HWE was discovered for this gene variant in both groups; χ^2^ values with 1 degree of freedom for the case and control groups were 2.326 and 2.880 respectively (*P* > 0.05). Analysis of the rs1801276 revealed a significant difference between the cases and controls. In comparison to the rs1801276 ‘CC’ genotype, the carriers of the "GG+GC" genotype occurred less frequently in the NAFLD patients than the controls even after adjustment for confounding factors such as age and BMI (*P* = 0.041; OR = 0.60, 95% CI = 0.45-0.93). In addition, the "G" allele of the *IRS1* rs1801276 variant was significantly less frequent in the patients than the controls too (*P* = 0.045; OR = 0.69, 95% CI = 0.58-0.91).

**Table 2:** Distribution of insulin receptor substrate 1 (*IRS1*) gene rs1801276 variant in nonalcoholic fatty liver disease (NAFLD) and control groups^a^

Gene (polymorphism)		Controls (n = 127)	Patients (n = 123)	OR (95% CI) P-value^b^
*IRS1* (rs1801276)				
Genotype-wise comparison				
	CC	88 (69.3)	100 (81.3)	1.0 (reference)
	CG	32 (25.2)	20 (16.3)	0.75 (0.61-4.69) 0.642
	GG	7 (5.5)	3 (2.4)	0.81 (0.52-4.40) 0.619
	CG and GG	39 (30.7)	23 (18.7)	0.60 (0.45-0.93) 0.041
	GG versus others	7 (5.5)	3 (2.4)	0.85 (0.49-4.07) 0.833
Allele-wise comparison				
	C	208 (81.9)	223 (90.7)	1.0 (reference)
	G	46 (18.1)	23 (9.3)	0.69 (0.58-0.91) 0.045

a Variables presented as number (%); The Hardy-Weinberg equilibrium for the rs1801276 variant was verified using chi-square (χ2) test. To compare allele and genotype frequencies between the control and case groups χ2 and logistic regression analysis were applied respectively. Logistic regression analysis was also used for adjusting confounding factors.

b Adjusted for age, body mass index, sex, smoking status, systolic blood pressure, and diastolic blood pressure in genotype-wise comparisons.

The possible relationships between the *IRS1* gene rs1801276 variant with BMI, systolic blood pressure, diastolic blood pressure, hypertension, diabetes, aspartate aminotransferase, alanine aminotransferase, gamma glutamyl transferase, steatosis, necroinflammation, and fibrosis in 123 NAFLD patients were also examined; no significant association was found (*P* ≥ 0.05).

## DISCUSSION

The potential association of the *IRS1* gene rs1801276 polymorphism with NAFLD risk was explored in the current study. We found for the first time that the *IRS1* rs1801276 "GG+GC" genotype compared to the "CC" genotype decreases the risk of NAFLD by 40 percent. Consistently, the *IRS1* rs1801276 "G" allele was under-represented in the cases with NAFLD too.

NAFLD is a multifactorial metabolic disorder which is derived from the coexistence of genetic and environmental factors. The identification of genes and polymorphisms underlying susceptibility to NAFLD and its clinical characteristics may help us to understand its pathophysiology and thus might afford the development of therapies. However, discovering these genetic variations is not as easy as it may appear due to the fact that contradictory results are not very rare in genetic association studies owing to differences in genetic background, genotyped markers, disease definition, diet, lifestyle, and even statistical methods (16-18). In light of its role in insulin signaling cascade, IRS1 may be involved in NAFLD pathogenesis. The human *IRS1* gene – a highly polymorphic gene with hundreds of SNPs – is located in the chromosome region of 2q36-37. The IRS1 and its pathway have a broad spectrum of biological functions, so any defects in them can lead to metabolic disorders such as IR and obesity that play a key role in NAFLD pathogenesis. In our previous study (13), no significant association was found between the *IRS1* gene Gly972Arg polymorphism and NAFLD risk. The present research, however, suggested that the Pro512Ala or rs1801276 variant of this gene might contribute to NAFLD. It appears that the "GG+GC" genotype and the "G" allele of this polymorphism to be markers of decreased NAFLD susceptibility. Perhaps the missense variant of the Pro512Ala per se not to be functional, and it is only a marker that has a strong linkage disequilibrium (LD) with the truly causal SNP. The other possibility is that the "C" allele of rs1801276 variant compared with the "G" allele might have a lesser IRS1 activity and ability to bind with PI3K, leading to a decrease in the activity of IRS1-PI3K pathway and finally increasing the risk of NAFLD. INSR signaling activity is downregulated in NASH and restoring it can be a reasonable target of pharmacological treatment in NAFLD. Likewise, IRS1 activation by inducing the PI3K-Akt signal transduction increases hepatic glucose uptake, glycogen synthesis, insulin sensitivity, and decreases lipogenesis, which in turn might ultimately attenuate NAFLD. Interestingly, treatment with Silibinin which is a hepato-protective agent ameliorates steatosis and IR partly through regulation of IRS1-PI3K-Akt signaling (19,20). Consistent with our findings, prior investigations have shown that other *IRS1* gene polymorphisms influence glucose intolerance, the survival of pancreatic β-cells, insulin secretion, serum insulin levels, insulin action, hyperinsulinemia, IR, T2DM, dyslipidemia, obesity, and hepatic fibrosis severity (9,11,12,19,21-24). Moreover, the genomic region ∼500 kb upstream of the *IRS1* gene is involved in IR, T2DM, and adverse lipid profile (25). Lastly, impaired glucose tolerance, IR, and resistance to IGF1 can be observed in mice with defect in *IRS1* gene (26,27). Accordingly, it is sensible to postulate that *IRS1* gene might participate in the development of NAFLD somehow, although its mechanism needs to be elucidated.

Several limitations of our study merit to be considered: (a) The sample size was relatively small due largely to limited funding and using liver biopsy, and thus the findings require further validation in larger sample groups. (b) Regarding budget limitations, circulating levels of insulin and glucose were not measured too, so we were unable to calculate IR. Notwithstanding limitations, the current study had some strengths that should be considered too. First, its design was good and we did multicenter research. Second, instead of using ultrasonography, the gold standard method for confirming NAFLD diagnosis (liver biopsy) was applied. Third, this report proposed some novel and interesting findings that were in accordance with previous literature too.

In conclusion, in summary, this is the first study showing that the *IRS1* rs1801276 "GG+GC" genotype and "G" allele have a protective effect for susceptibility to NAFLD. Although this observation supports prior publications, more investigation in other populations of different ethnic origins is needed.
